# Evaluating the effect of probiotic supplementation in the form of mouthwash along with scaling and root planing on periodontal indices in patients with stage III and grade A generalized periodontitis: A randomized clinical trial

**DOI:** 10.34172/japid.2020.013

**Published:** 2020-12-09

**Authors:** Shima Ghasemi, Amir Reza Babaloo, Behnam Mohammadi, Mahdieh Esmailzadeh

**Affiliations:** ^1^Department of Prosthodontics, Faculty of Dentistry, Tabriz University of Medical Sciences, Tabriz, Iran; ^2^Department of Periodontics, Faculty of Dentistry, Tabriz University of Medical Sciences, Tabriz, Iran; ^3^Private Practice, Tabriz, Iran; ^4^Dentistry Student, Faculty of Dentistry, Tabriz University of Medical Sciences, Tabriz, Iran

**Keywords:** Periodontitis, Probiotic, Root planning

## Abstract

**Background:**

Periodontal disease is a chronic polymicrobial infectious condition. Non-surgical treatments, including scaling and root planing (SRP) with or without adjunctive treatments, are among the recommended treatment options for this condition. This study investigated the effect of probiotic supplementation in the form of mouthwash with SRP on periodontal indices in patients with stage III grade A generalized periodontitis.

**Methods:**

Thirty-six patients were randomly assigned to two groups (n=18) and received SRP treatment along with a placebo in one group and probiotic supplementation in the other. After SRP, the test group used daily probiotics for 20 days. The control group subjects were treated only with SRP and placebo mouthwash. Periodontal indices were determined at three time intervals: at baseline and after one and three months. The data were analyzed using SPSS 17. P<0.05 was considered statistically significantd.

**Results:**

There were significant differences in BOP levels in both the test and control groups between different intervals, with no significant difference between the groups. The significance of changes in the CAL and PI indices were similar to those in BOP. There were significant differences in PD levels between the groups after one and three months using the mouthwash. There were also significant differences between the PD values at different intervals in both groups.

**Conclusion:**

This study’s results showed that probiotic supplementation as a mouthwash, along with SRP, had a positive effect on periodontal indices in patients with stage III and grade A generalized periodontitis.

## Introduction

Periodontal disease is a chronic polymicrobial infectious condition with persistent inflammation, connective tissue disruption, and alveolar bone resorption.^1^ Periodontitis is a multifactorial disease that involves interaction between environmental, host, and microbial factors.^1^ Non-surgical treatment includes scaling and root planing (SRP) with or without adjunctive treatments and medications such as antimicrobials, antiseptics, and probiotics with different delivery forms as tablets, lozenges, or mouthwashes.^2^ Studies have shown that it is not possible to eliminate all the microbial plaques and bacteria from the infected root surfaces, and complete elimination of the underlying gingival microbial layer requires an appropriate antimicrobial concentration with a proper duration. Therefore, it seems necessary to use adjunctive treatment in addition to standard SRP treatments for a successful outcome.^3^ Different studies have used different clinical indices to investigate the effect of these adjunctive therapies. Sulcus bleeding index is one of the indices designed to investigate gingival bleeding.^4^ The amount of plaque, calculus, inflammation, gingival bleeding, the periodontal pocket depth, and the rate of alveolar bone resorption have also been investigated in such studies.^5^ SRP is the primary treatment modality for periodontal disease.^6^ The chief aim of this treatment is to mechanically debride bacterial plaque and eliminate bacterial products and processes from the root surface and the periodontal pockets.^7^ The host and the bacteria are both critical in inducing periodontal diseases. The periodontopathogenic bacteria, the absence of useful bacteria, and the host susceptibility are the principal causes of periodontal disease.^8^ Various primary treatment modalities are available, including mechanical approaches such as surgical or non-surgical treatments for periodontal disease.^2^ Lily and Steele^3^ first introduced the term probiotics as the substances produced by microorganisms that promote other microorganisms’ growth and proliferation. The WHO and the Food and Drug Administration (FDA) proposed the current definition of probiotics. They defined probiotics as live microorganisms that are beneficial to the host’s health when consumed in sufficient amounts. Probiotics improve human health by inhibiting or reducing the pathogenic microorganisms. The most common probiotic bacteria are *Lactobacilli*^9^ and *Bifidobacteria*.^10^ Previous studies have reported different results on the effects of various probiotic species on oral health and lowering the levels of *Streptococcus mutans* in the saliva, limiting the metabolic opportunities for the pathogens by occupying microbial niches with normal and non-pathogenic microflora. Probiotics inhibit the colonization of periodontal pathogens through various mechanisms. Therefore, they reduce the counts of pathogenic bacteria.^1^ The probiotic bacteria should be able to adhere to tooth surfaces and enter the dental biofilm to exert a positive effect on the oral cavity. They should also be able to compete with and prevent the proliferation of cariogenic bacteria.^11^

Only a few studies have investigated probiotics’ role in treating periodontitis. This study investigated the effect of probiotic mouthwash along with SRP on periodontal indices in patients with stage III grade A generalized periodontitis. The present study investigated the effect of probiotic supplementation in the form of mouthwash on the clinical indices of probing depth (PD), bleeding on probing (BOP), clinical attachment loss (CAL), and plaque index (PI).

## Methods

The Ethics Committee of Tabriz University of Medical Sciences approved the present randomized, double-blind, placebo-controlled, parallel study under the code IR.TBZMED.VCR.REC.1397.058. All the procedures of the study conformed to the Declaration of Helsinki, as revised in 2008. This clinical trial was registered in the Iranian Registry of Clinical Trials and approved by the International Committee of Medical Journal Editors (ICMJE). The registration date is 2019/03/27, and the *Iranian Registry of Clinical Trials (*IRCT) registration number is IRCT20180630040290N2.

The present study evaluated the effect of the topical use of probiotic supplements, along with SRP in patients with stage III grade A generalized periodontitis in three months in the Department of Periodontics, Faculty of Dentistry, Tabriz University of Medical Sciences. The sample size was calculated at 28 samples using the results of a study by Penala.^12^ To increase the study’s validity, and due to possible sample loss, the sample size was increased by 30%. Finally, 36 samples (n=18 in each group) were included in the study. The participants were selected from the patients referred to the Department of Periodontics, Faculty of Dentistry, Tabriz University of Medical Sciences, from September 2018 to November 2018.

The inclusion criteria consisted of otherwise healthy subjects with an age range of 25–59 years and a mean age of 45.3 years, a definitive diagnosis of periodontitis, at least four teeth with a PD of ≥5 mm, and a CAL of ≥4 mm. The exclusion criteria consisted of patients with any systemic condition possibly affecting the periodontium that could change the course of periodontitis, patients with allergy to probiotic supplements, patients treated with antibiotics in the past three months, patients undergoing any periodontal treatment in the past six months, pregnant and lactating women, patients with a history of smoking or any other habit, and patients with a history of allergy to lactate products. At baseline, the clinical parameters, including PD and CAL in six sites per tooth and bleeding index (BI), were determined using a UNC-15 probe. Also, the PI index was measured from the baseline to the end of the study. First, each patient underwent a complete SRP procedure with manual and ultrasonic tools. After the SRP, the patients were given oral hygiene instructions. Computer-generated random numbers were used to assign the patients to the test and control groups (n=18).

The probiotic mouthwash used in the study was in the form of a Prokid capsule (15×10^9^ probiotic units per capsule), which contained a combination of bacterial strains, i.e., *Bifidobacteriumlactis*, *Lactobacillus acidophilus*, *Bifidobacterium bifidum*, *Lactobacillus rhamnosus*, which were purchased from Gostaresh Milad Pharmed Co. After the SRP procedure, the test group patients used the probiotics daily for 20 days by dissolving two probiotic capsules in 250 mL of water and gargling it for 60 seconds to increase its local effect on the gingival tissue.^12^ The control group patients were treated only with SRP and a placebo mouthwash of pure water. The subjects were asked to refrain from eating, drinking, chewing, brushing, and rinsing their oral cavities for two hours before each appointment. Probiotic and placebo capsules were labeled in the same containers and given A and B codes. Since probiotics and placebo supplements were placed in the same capsule, it was evident that the patient and the clinician were not aware of the type of medication taken. To ensure allocation, the patient codes were preserved by a researcher based on the serial number. The primary results, including BOP,^13^ PD,^14^ CAL,^15^ and PI,^16^ were measured from the baseline to the end of the study.

### Data analysis

The results were reported as mean ± standard deviations and frequencies (percentages). Repeated-measures ANOVA was used to compare PD, CAL, and PI in the two groups, using SPSS 17; the significance level was defined at P<0.05.

## Results

This clinical trial was performed on 36 patients with a mean age of 44.58 years, divided into the control (mean age of 44.35 years) and test (mean age of 44.81 years) groups randomly, with 60% males and 40% females in the control group and 55% males and 45% females in the test group, with no significant differences between the two groups in age and gender (P>0.05). Periodontal indices were measured at three intervals in both groups: baseline and after one and three months. These indices included (BOP), (PI), (PD), and (CAL). Table 1 presents the descriptive statistics for periodontal indices. As the data show, there was a difference of 1.39 units between BOP levels in the two groups at baseline, which was higher in the test group, with no significant difference (P=0.601). After using the mouthwash for one month, the difference reached 5.21 units, which was higher in the control group and was reported to be insignificant (P=0.087). Moreover, after three months, the difference between the two groups decreased to 3.98 units, which was higher in the control group, with no significant difference between the two groups (P=0.245). The repeated-measures ANOVA was applied separately in each group to compare the BOP index at different time intervals. According to Tables 2 and 3, the results showed significant differences between BOP values ​​at different intervals in both the control and test groups (P<0.001).

**Table 1:** Descriptive statistics for the clinical indices of probing depth (PD), bleeding on probing (BOP), gingival attachment loss (CAL), and plaque index (PI) at different intervals in both the control and test groups

**Periodontal index**	**n**	**control**	**test**	**P-value**
**BOP baseline**	18	47.55±8.14	48.95±7.73	0.601
**BOP after 1 month**	18	35.77±7.68	30.56±9.89	0.087
**BOP after 3 months**	18	23.45±9.17	19.46±10.97	0.245
**PI baseline**	18	48.72±7.71	50.76±7.87	0.438
**PI after 1 month**	18	33.34±10.09	30.57±11.84	0.456
**PI after 3 months**	18	20.11±7.54	19.59±10.79	0.868
**PD baseline**	18	5.78±0.57	5.65±0.57	0.509
**PD after 1 month**	18	5.33±0.67	4.86±0.58	**0.031** ^*^
**PD after 3 months**	18	4.97±0.68	4.29±0.71	**0.006** ^*^
**CAL baseline**	18	5.33±0.69	5.31±0.58	0.949
**CAL after 1 month**	18	4.96±0.71	4.67±0.63	0.204
**CAL after 3 months**	18	4.69±0.69	4.25±0.76	0.075

*significant difference

**Table 2:** Descriptive statistics for the clinical indices at different intervals in the control group

Clinical Index	Baseline	After 1 month	After 3 months	P-value
BOP	47.55±8.14	35.77±7.68	23.45±9.17	<0.001*
PI	48.72±7.71	33.34±10.09	20.11±7.54	<0.001*
PD	5.78±0.57	5.33±0.67	4.97±0.68	<0.001*
CAL	5.33±0.69	4.96±0.71	4.69±0.69	<0.001*

*significant difference

**Table 3:** Descriptive statistics for the clinical indices at different intervals in the test group

**Clinical Index**	**Baseline**	**After 1 month**	**After 3 months**	**P-value**
**BOP**	48.95±7.73	30.56±9.89	19.46±10.97	<0.001*
**PI**	50.76±7.87	30.57±11.84	19.59±10.79	<0.001*
**PD**	5.65±0.57	4.86±0.58	4.29±0.71	<0.001*
**CAL**	5.31±0.58	4.67±0.63	4.25±0.76	<0.001*

*significant difference

According to Table 1, at baseline, the mean PI in the control group was 5.78±0.57, which was the highest; after three months, PI was reported at 4.29±0.71 in the test group, which was the lowest rate. There was a difference of 2.03 units between the PI levels in the two groups at baseline, which was higher in the test group, with no significant difference (P=0.438). After using the mouthwash for one month, the difference reached 2.76 units, which was higher in the control group, with no significant difference (P=0.456). Also, after three months, the difference between the two groups decreased to 0.52, which was higher in the control group, with no significant difference between the two groups (P=0.868). The repeated-measures ANOVA was applied separately in each group to compare the PI index at different time intervals. Tables 2 and 3 show a significant difference between PI values ​​at different intervals in both the control and intervention groups (P<0.001).

Descriptive statistics for the pocket depth index are presented in Table 1. As the data show at baseline, the mean PD in the control group was 5.78±0.57, which was the highest; after three months, PD was reported at 4.29±0.71 in the test group, which was the lowest rate. There was a difference of 0.12 units between the PD levels in the two groups at baseline, which was higher in the control group, with no significant difference (P=0.509). After using the mouthwash for one month, this difference reached 0.47 units, which was again higher in the control group, and the difference was significant (P=0.031). After three months, the difference between the two groups increased to 0.68 units, which was again higher in the control group, and the difference between the two groups was significant (P=0.006). The repeated-measures ANOVA was applied separately in each group to compare the PD index at different time intervals. According to Tables 2 and 3, there was a significant difference between PD values ​​at different intervals in both the control and test groups (P<0.001).

Descriptive statistics related to the CAL index are presented in Table 1. Based on the baseline data, the mean CAL in the control group was 5.33±0.57, which was the highest. After three months, CAL was reported at 4.25±0.76 in the test group, which was the lowest rate. There was a difference of 0.01 units between the CAL values between the two groups at baseline, which was higher in the control group; however, the difference not significant (P=0.949). After using the mouthwash for one month, this difference reached 0.29 units, which was higher in the control group, but the difference was not significant (P=0.204). After three months, the difference between the two groups increased to 0.444, which was again higher in the control group, with no significant difference between the two groups (P=0.075). The repeated-measures ANOVA was applied separately in each group to compare the PI index at different time intervals. Tables 2 and 3 show a significant difference between CAL values ​​at different intervals in both the control and test groups (P<0.001).

## Discussion

Periodontal diseases are common multifactorial conditions in different communities.^17^ One of the most important factors responsible for these diseases is the disruption of the oral cavity microbial flora.^18^ Due to the importance of eliminating pathogenic strains in different types of periodontitis, current therapies, including non-surgical treatments such as SRP with or without supplemental treatments, are among the recommended options for recovery.^19^ Despite its various benefits, antibiotic therapy leads to the development of resistant strains, and if inappropriate antibiotics are selected, disease recurrence is not unexpected.^20^ Other methods, despite their various benefits, are costly, and their success depends on controlling pathogenic bacteria and environmental factors. Therefore, probiotics appears to be a necessity in the treatment of various diseases, including periodontal diseases.^21^ Probiotics are living bacteria that affect the host by improving the microbial balance in their body.^21^ According to the FDA, probiotics are living microorganisms with health benefits for the host in sufficient amounts.^22^ Considering the discrepancies about the effects of different probiotic strains on oral health, this study investigated the effect of probiotic supplementation as a mouthwash along with SRP on periodontal indices in patients with stage III grade A generalized periodontitis. The results showed that in both the test and control groups, the periodontal indices exhibited significant changes over time. However, the test group exhibited a higher rate of change. The current results are consistent with previous studies in this field.

Penala et al^12^ studied the effect of a probiotic mouthwash containing *Lactobacillus salivarius* and *Lactobacillus reuteri* on changing the periodontal indices of patients with chronic periodontitis and reported that the application of probiotics in the mouthwash and subgingival form along with SRP treatment for 15 days could significantly reduce the plaque index and BOP in three months. The study also showed that both SRP + placebo and SRP + probiotic treatments significantly reduced the mean PD and CAL in patients, with no significant difference between the two groups. The researchers divided the patients’ periodontal pockets into two subgroups, 4–6 mm (medium depth) and deep (>7 mm). Data analysis showed that probiotics significantly affected the depth of medium pockets, with no significant effect on deep pockets. These findings are consistent with our study in terms of significant changes in both the test and control groups. The results also showed that a more detailed study of pocket depths could lead to a greater understanding of how probiotics affect the pocket depth. In our study, changes in pocket depth were significant between the two test and control groups; however, in the study by Penala et al, these changes were not significant. The difference between the results could be attributed to a higher mean of patients’ pocket depths and a wider variety of bacteria, which reduces the therapeutic effects of probiotics. Ince et al^23^ studied 30 patients 35–50 years of age with chronic periodontitis and reported that the use of *L. reuteri* probiotics along with SRP treatment for three weeks could significantly improve PI, GI, and BOP indices. Also, the results showed that probiotics decreased the matrix metalloproteinase (MMP-8) levels in the *gingival crevicular fluid*and significantly increased the levels of tissue inhibitor of metalloproteinase (TIMP-1) after 180 days, consistent with the present study in terms of significant changes in BOP and PI.

Szkaradkiewicicz et al^24^ studied 38 patients aged 31–46 years with chronic periodontitis. The patients underwent treatment with SRP along with *L. reuteri* probiotics in pill form over a two-week interval. The results showed that probiotics significantly reduced the pocket depths and CAL. In addition, these researchers reported that pro-inflammatory cytokines, such as *Tumor necrosis factor*-α (TNF-α), *Interleukin*-1β (IL-1β), and *Interleukin*-17 (IL-17), decreased significantly in patients receiving probiotics. These findings, too, are consistent with the current results. However, it should be noted that the study used probiotic pills; therefore, it could be concluded that regardless of the form of probiotics, it could achieve the desired therapeutic esults.A study by Vicario et al^25^ on *L. reuteri* in tablet form for 30 days in patients aged 44–65 years with chronic periodontitis showed that non-smokers and patients with initial-to-moderate chronic periodontitis showed a faster and better response to probiotics use. PI, BOP, and PD were among the indices, which improved significantly in these patients. This study showed that tobacco use interferes with the effect of probiotics; therefore, it could be considered as a confounding factor in probiotic studies.

Teughles et al^10^ studied 30 patients >35 years of age with chronic periodontitis and reported that *L. reuteri* could significantly reduce medium and deep pockets in 12 weeks. Further investigations also showed that *P. gingivalis* levels significantly decreased due to the use of probiotics, consistent with the present study, and also indicating that by increasing the duration of probiotic use, it is possible to decrease pocket depths.

Riccia et al^26^ studied the *Lactobacillus brevis* probiotic effect over a short period of four days. The results showed that the patients’ clinical parameters, including gingivitis, bleeding on probing, and plaque index, significantly decreased in probiotic users. These results are consistent with the current study, indicating that in cases where long-term probiotic use is not possible, their use is still justified and can lead to optimal therapeutic results in a short time.

## Conclusion

The present study showed that probiotic supplementation as a mouthwash, along with SRP, positively affected periodontal indices in patients with stage III and grade A generalized periodontitis, accelerating the treatment process and significantly reducing pocket depths. Therefore, the use of probiotics as a mouthwash is recommended in clinical procedures.

## Ethics Approval

The ethics approval for this study was obtained from the Ethics Committee of Tabriz University of Medical Sciences.

## Authors’ Contributions

The study was planned and designed by S.G. and AR. B.B.M and A.R.B conducted the clinical experiments and contributed to data acquisition. The statistical analyses and interpretation of data were carried out by B.M. and S.G. M.E contributed to the literature review and manuscript preparation and manuscript editing. All the authors have read and approved the final manuscript.

## Conflicts of Interests

The authors declare no conflicts of interest.

## Data Sharing Statement

The data that support the findings of this study are available from the corresponding author, upon reasonable request.
